# Brucine Suppresses Vasculogenic Mimicry in Human Triple-Negative Breast Cancer Cell Line MDA-MB-231

**DOI:** 10.1155/2019/6543230

**Published:** 2019-01-06

**Authors:** Meng-Ran Xu, Peng-Fei Wei, Ming-Zhu Suo, Yi Hu, Weiping Ding, Li Su, Yao-Dong Zhu, Wan-Ji Song, Guan-Hao Tang, Mei Zhang, Ping Li

**Affiliations:** ^1^Department of Chinese Integrative Medicine Oncology, The First Affiliated Hospital of Anhui Medical University, Hefei, Anhui 230022, China; ^2^The CAS Key Laboratory of Innate Immunity and Chronic Disease, Innovation Center for Cell Signaling Network, School of Life Sciences and Medical Center, University of Science and Technology of China, Hefei, Anhui 230027, China; ^3^Hefei National Laboratory for Physical Sciences at Microscale, Hefei, Anhui 230027, China; ^4^Center for Biomedical Engineering, Department of Electronic Science and Technology, University of Science and Technology of China, Hefei, Anhui 230027, China

## Abstract

Vasculogenic mimicry (VM) with the pattern of endothelial independent tubular structure formation lined by aggressive tumor cells mimics regular tumor blood vessels to ensure robust blood supply and correlates with the proliferation, invasion, metastasis, and poor prognosis of malignant tumors, which was demonstrated to be a major obstacle for resistance to antiangiogenesis therapy. Therefore, it is urgent to discover methods to abrogate the VM formation of tumors, which possesses important practical significance for improving tumor therapy. Brucine is a traditional medicinal herb extracted from seeds of Strychnos nux-vomica L. (Loganiaceae) exhibiting antitumor activity in a variety of cancer models. In the present study, the effect of brucine on vasculogenic mimicry and the related mechanism are to be investigated. We demonstrated that, in a triple-negative breast cancer cell line MDA-MB-231, brucine induced a dose-dependent inhibitory effect on cell proliferation along with apoptosis induction at higher concentrations. The further study showed that brucine inhibited cell migration and invasion with a dose-dependent manner. Our results for the first time indicated that brucine could disrupt F-actin cytoskeleton and microtubule structure, thereby impairing hallmarks of aggressive tumors, like migration, invasion, and holding a possibility of suppressing vasculogenic mimicry. Hence, the inhibitory effect of brucine on vasculogenic mimicry was further verified. The results illustrated that brucine significantly suppressed vasculogenic mimicry tube formation with a dose-dependent effect indicated by the change of the number of tubules, intersections, and mean length of tubules. The in-depth molecular mechanism of vasculogenic mimicry suppression induced by brucine was finally suggested. It was demonstrated that brucine inhibited vasculogenic mimicry which might be through the downregulation of erythropoietin-producing hepatocellular carcinoma-A2 and matrix metalloproteinase-2 and metalloproteinase-9.

## 1. Introduction

Solid malignant tumors possessing the characteristics, such as rapid growth and metastasis, a high tumor grade, and bidirectional differentiation or uncommitted differentiation status, could build tubular structures lined by aggressive tumor cells and mimic regular blood vessels to meet their requirement for masses of oxygen and nutrients [1–4]. The tumors creating this endothelial independent vascularization with the CD34 negative and periodic acid Schiff (PAS) positive patterns are defined to have vasculogenic mimicry (VM) [2, 3]. VM has also been demonstrated to be involved in the proliferation, invasion, metastasis, and tumor poor prognosis of malignant tumors [2, 4]. The clinical strategy targeting intratumor vascular endothelial cells is always ineffective for the therapy of the aggressive tumors possessing VM capacity [5–8], indicating that the development of VM is possibly a major obstacle for resistance to antiangiogenesis therapy [9]. Therefore, it is imperative to discover methods to overcome the VM capacity of tumors, which have important practical significance for improving the therapeutic effect on tumors.

**Figure 1:**
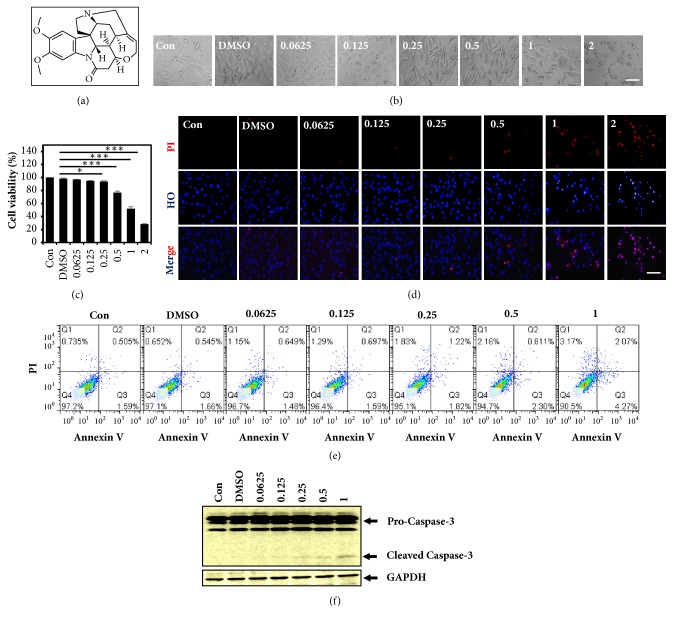
The effect of brucine on MDA-MB-231 cell viability and apoptosis. The chemical structure of brucine was presented here (a). After MDA-MB-231 cells treated with the vehicle control (DMSO) or 0.0625, 0.125, 0.25, 0.5, 1, or 2 mM brucine for 24 h, the cell viability was evaluated by MTT assay (b), and bright field images (c) and Hochest33342/PI double staining photographs (d) of MDA-MB-231 cells were captured under microscopy. After MDA-MB-231 cells treated with the vehicle control (DMSO) or 0.0625, 0.125, 0.25, 0.5, or 1 mM brucine for 24 h, cell apoptosis was detected with the Annexin V-FITC Apoptosis Detection Kit (e); the apoptosis-related protein caspase-3 cleavage was measured by western blot assay (f). The scale bar is of 100 *μ*m (mean ± s.e.m., n = 3, *∗*p < 0.05, and *∗∗∗*p < 0.001.)

Triple-negative breast cancer (TNBC) is one of the most controversial women malignancies, which is always lethal in most clinical cases [10]. Vasculogenic mimicry in TNBC was reported to be reinforced by antiangiogenic treatment and promoted tumor progression [7, 8], which is not beneficial for tumor therapy. Brucine, an indole alkaloid (Figure 1(a)) extracted from seeds of Strychnos nux-vomica L. (Loganiaceae), is a traditional medicinal herb native to India, Southeast Asia, and northern Australia [11]. Sarita Saraswati et al. reviewed that brucine could induce anti-inflammation, antitumor, and antiproliferation effect and be used for the treatment of analgesia, diabetes, anemia, and gonorrhea [11, 12]. Importantly, the inhibitory effect of brucine on tumor angiogenesis was suggested [11, 12]. However, the regulatory effect of brucine on VM and its related molecular mechanisms are rarely reported.

In this study, we observed the effect of brucine on VM, explored the possible roles of cell migration and invasion inhibition and cytoskeleton disruption, measured the change of the protein levels of VM-related molecular markers, and discovered the potential of brucine as a vasculogenic mimicry inhibitor for cancer treatment.

## 2. Materials and Methods

### 2.1. Materials

Brucine (399027) and dimethyl sulfoxide (DMSO, D2650) were purchased from Sigma-Aldrich (St. Louis, MO). Standard stock solutions of brucine (500 mM) were prepared by dissolving brucine with DMSO. PAS staining Kit (G1360) was from Beijing Solarbio Science & Technology Company. Alexa Fluor™ 488 Phalloidin (A12379) and Tubulin-*β* Rabbit Polyclonal Antibody (RB-9249-P0) were purchased from Thermo Fisher Scientific Company. 4′,6-diamidino-2-phenylindole (DAPI, C1002), Hoechst 33342 (C1022), propidium iodide (PI, ST511), and Annexin V-FITC Apoptosis Detection Kit (C1063) were purchased from Beyotime Company (Shanghai, China). Thiazolyl blue tetrazolium bromide (MTT, T0793) was purchased from Bio Basic. Falcon® Permeable Support for 24-well plate with 8.0 *μ*m Transparent PET Membrane (353097), Falcon® 24-well TC-treated Cell Polystyrene Permeable Support Companion Plate (353504), and Corning® Matrigel® Basement Membrane Matrix (356234) were purchased from Corning. MMP-2 Antibody (sc-13594), MMP-9 Antibody (sc-21733), and Rhodamine conjugated Donkey anti-rabbit IgG-R (sc-2095) were purchased from Santa Cruz Biotechnology (Texas, USA). Human EphA2 Antibody (AF3035) was purchased from R&D Systems (Minneapolis, MN). Antibody Anticleaved caspase-3 (9665) was purchased from Cell Signaling Technology. Anti-GAPDH antibody (AB9132) was purchased from Merck Millipore (Darmstadt, Germany). HRP-conjugated Goat Anti-Rabbit IgG (H+L) (SA00001-2) was purchased from Proteintech. HRP-conjugated anti-mouse antibody (W4021) and Donkey Anti-Goat IgG (V8051) were purchased from Promega (Madison, USA). Enhanced chemiluminescence (ECL) kits were from Biological Industries (Kibbutz beit Haemek, Israel). Crystal violet (KGA229) was from Nanjing KeyGen Biotech Company (Nanjing, China).

### 2.2. Cell Lines and Cell Culture

Human breast cancer cell MDA-MB-231 was obtained from the Cell Bank of Chinese Academy of Sciences (Shanghai, China). MDA-MB-231 was maintained in Leibovitz's L-15 medium (LA9510, Solarbio) supplemented with 10% fatal bovine serum (BI, USA), 100 U/mL penicillin, and 100 *μ*g/mL streptomycin (Sigma-Aldrich, St. Louis, MO) at 37°C in a cell culture incubator with atmospheric air.

### 2.3. Cell Viability Assay

Cell viability was determined by MTT assay. Briefly, MDA-MB-231 cells, seeded in 96-well plates at a density of approximately 10,000 cells per well, were treated with different concentrations of brucine for 24 h. After that, MTT was added to each well at a final concentration of 0.5 mg/mL. After 3 h incubation at 37°C, remove the medium supernatant and 100 *μ*L of DMSO was added to each well, and then plates were agitated for 1 min. The optical density (OD) at 570 nm was measured with a spectrophotometer (Elx800, BioTek, Winooski, VT, USA). The experiment was carried out three times.

### 2.4. Hoechst 33342/PI Double Staining

MDA-MB-231 cells, related to different concentrations of brucine for 24 h, were stained with Hoechst 33342 (20 *μ*g/mL) and PI (10 *μ*g/mL) for 10 min and then examined under fluorescence microscopy (Olympus IX71).

### 2.5. Cell Apoptosis

Apoptosis detection was performed with the Annexin V-FITC Apoptosis Detection Kit (C1063) according to the manufacturer's instructions and analyzed by flow cytometry (FACScan, BD Biosciences).

### 2.6. Scratch Wound Healing Assay

The method was previously described [13]. Briefly, a scratch wound was performed on the 90% confluent MDA-MB-231 cell monolayer using a 200 ml yellow tip. After that, it was further incubated in the presence of brucine and photographed at the indicated time periods under an inverted microscopy (Olympus IX71). Wound closure was measured with ImageJ software.

### 2.7. Cell Invasion Assay

Transwell chambers (8 *μ*m pore-size, Corning Company, USA) were used here. Matrigel (356234, Corning Company, USA) was dissolved at 4°C overnight, diluted with serum-free medium (1: 8), added to the upper chambers with 50 *μ*L in each well and balanced in an incubator to form a gel for 30 min at 37°C. Various concentrations of brucine (0.0625, 0.125, 0.25, 0.5, and 1 mM) were exposed to the cells for 24 h before seeding. Then MDA-MB-231 cells (1 × 10^5^ cells/mL) were seeded into the upper chambers containing serum-free Leibovitz's L-15 medium, and a medium containing 10% FBS was added to the lower chambers. After incubating for 24 h at 37°C in atmospheric air, the noninvasive cells that remained on the upper side of the insert membranes were removed with a cotton swab, and the invaded cells on the lower side of the insert membranes were fixed and stained with 4% paraformaldehyde and then stained with 10% crystal violet. The stained cells were observed and counted under a microscope. Data were collected from three independent experiments.

### 2.8. Vascular Mimicry Formation

24-well cell culture plate wells were evenly coated with Basement Membrane Matrix Matrigel (356234, Corning Company, USA), which was allowed to solidify at 37°C for 30 min. Then, the MDA-MB-231 cells having been treated with various concentrations of brucine (0.0625, 0.125, 0.25, 0.5, and 1 mM) were added (1 × 10^5^ cells/mL) onto the surface of the matrigel and incubated at 37°C for the indicated time periods. The vascular mimicry formation photographs were captured under the microscopy (Olympus IX71), and the number of tubules plus intersections and mean length of tubules were evaluated using ImageJ software.

### 2.9. Observation of the Cell Cytoskeleton by Laser Confocal Microscopy

After MDA-MB-231 cells grown on glass cover slips in 24-well cell culture plates for 24 h, they were then treated with brucine (0.5 and 1 mM) for 24 h. After that, MDA-MB-231 cells on the cover slips were fixed with 4% formaldehyde for 10 min at room temperature, permeabilized with 0.1% Triton X-100 for 3 min, and subsequently blocked with PBS containing 1% bovine serum albumin (BSA) for 1 h. The cells were incubated with tubulin-*β* antibody at 1:1000 dilution at room temperature for 1 h, washed with PBS, and incubated with Rhodamine conjugated Donkey anti-rabbit IgG-R (sc-2095) with 1:400 dilution at room temperature during hour for immunofluorescence staining of microtubules. The cells were stained with Alexa Fluor™ 488 Phalloidin with the working concentration 10^−8^ mol/L to indicate F-actin cytoskeleton. Cell nucleus was stained by DAPI with the working concentration 5 *μ*g/mL. All the photographs were captured under a confocal laser-scanning microscope (Zeiss LSM710).

### 2.10. Western Blot Assay

After harvesting via trypsinization, cell pellets were resuspended with the lysis buffer (0.5% Nonidet P-40, 10 mM Tris-HCl, 100 mM NaCl, pH 7.5) supplemented with a protease inhibitor cocktail (Sigma, P8340) on ice. Protein samples were homogenized with equal volume of 2 ×SDS sample buffer and heated to 100°C for 5 min, and each sample was then separated by 12% SDS-PAGE. Then, proteins were transferred to nitrocellulose membranes (Millipore, Bedford, MA, USA). After blocking with Tris-buffered saline containing 0.1% Tween-20 (TBST) and 5% nonfat dry milk at room temperature for 1 hour, the nitrocellulose membranes were incubated with different primary antibodies overnight at 4°C. Membranes were washed with TBST and incubated with HRP-conjugated second antibodies for 1 hour at room temperature. Finally, protein expressions were examined using an ECL Kit. Densitometry measurement was performed using ImageJ software.

### 2.11. PAS Staining of Vasculogenic-Like Networks In Vitro

MDA-MB-231 cells were fixed by 4% paraformaldehyde, stained by PAS stain according to the manufacturer's protocols and then observed under a phase contrast microscope (Olympus IX71).

### 2.12. Statistical Analysis

All data were obtained from three independent experiments and all values were represented as the means ± SD. Statistical analysis was performed using SPSS software (version 19.0). The results were subjected to one-way ANOVA using the Duncan test to analyze the difference among experimental groups. P-value less than 0.05 was considered as significant difference.

## 3. Results

### 3.1. Inhibitory Effect of Brucine on MDA-MB-231 Proliferation In Vitro

The molecular structure of brucine was showed in Figure 1(a). Herein, the inhibitory effect of brucine on MDA-MB-231 cells was firstly observed under microscope. The number of cells was significantly reduced at higher concentrations (1, 2 mM) after the treatment with brucine for 24 h (Figure 1(c)). In addition, it caused cell morphological changes with rounding and shrinking of cell shapes and gradual loss of their long spindle shape compared to control group cells (Figure 1(b)). The results of MTT assay showed that the absorption value of MDA-MB-231 cells treated with the vehicle control or 0.0625, 0.125, 0.25, 0.5, 1, or 2 mM brucine for 24 h was 98.200 ± 0.998, 0.972 ± 0.468, 94.737 ± 0.771, 93.80 ± 1.068, 76.749 ± 2.337, 52.038 ± 2.961, and 28.433 ± 0.484, respectively (Figure 1(c)). And the data were calculated from three independent experiments. The 50% inhibitory concentration (IC50) of brucine on MDA-MB-231 cells with 24 h treatment was 1.172 mM. These data showed that brucine treatment exhibited dose-dependent inhibitory effect on MDA-MB-231 cell growth. Herein, we used the doses below IC50 of brucine to optimize the following experiments.

### 3.2. Brucine Induces MDA-MB-231 Cell Apoptosis

In accordance with previous studies illustrated by brucine induced growth inhibition with concentration dependent manner, propidium iodide (PI) staining assay showed that brucine induced dose-dependent cell death with obvious increase at the higher concentrations (1, 2 mM) after treatment with brucine for 24 h (Figure 1(d)). Moreover, Annexin V/PI staining assay followed by FACS measurement illustrated that brucine caused cell apoptosis but with only 4.27% apoptosis at the concentration of 1 mM (Figure 1(e)). Western blot assay also showed that brucine induced cell apoptosis indicated by increased cleaved caspase-3 only at the higher concentrations (Figure 1(f)).

### 3.3. MDA-MB-231 Cell Migration and Invasion Inhibition by Brucine

**Figure 2:**
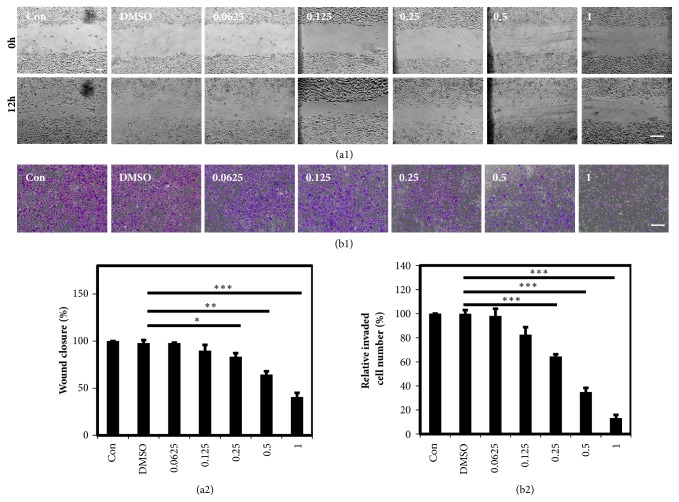
Depression of MDA-MB-231 cell migration and invasion by brucine. (a1-a2) The scratch wound healing assay indicated that brucine caused a dose-dependent suppression on MDA-MB-231 cell migration after the treatment with different concentrations of brucine for 12 h. (b1-b2) After MDA-MB-231 cells treated with brucine for 24 h, the invaded cell numbers were significantly reduced with a dose-dependent effect. The scale bar is of 100 *μ*m (mean ± s.e.m., n = 3, *∗*p < 0.05, *∗∗*p < 0.01, and *∗∗∗*p < 0.001.)

The migration (Figures 2(a1)-2(a2)) and invasion (Figures 2(b1)-2(b2)) of the MDA-MB-231 cells were significantly changed between control and DMSO groups.

### 3.4. The Effects of Brucine on the Cytoskeleton of MDA-MB-231 Cells

**Figure 3:**
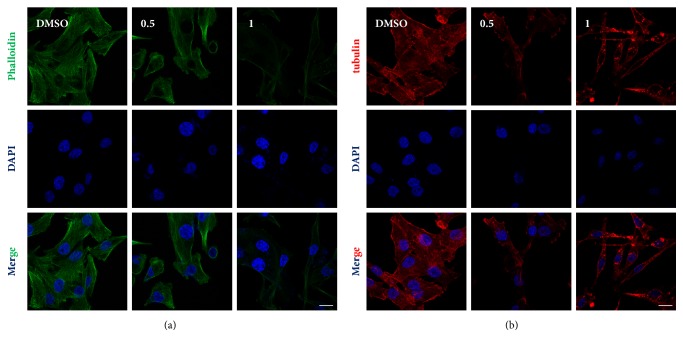
The aberrations of F-actin cytoskeleton and microtubule structure induced by brucine. After MDA-MB-231 cells treated with brucine (0.5; 1 mM) for 24 h, the disruption of F-actin cytoskeleton stained by Alexa Fluor™ 488 Phalloidin (a) and microtubule structure indicated by immunofluorescence staining (b) were observed under a confocal laser-scanning microscopy. Nuclei were counterstained with DAPI (blue). The scale bar is of 5 *μ*m.

Fluorescence-conjugated phalloidin was used to detect the F-actin cytoskeleton in the brucine treated or untreated MDA-MB-231 cells. Under the confocal microscope, F-actin was distinct in the control group, showing a compact and directional alignment with obvious fibrous tension. On the other hand, F-actin was loosely aligned after the treatment with brucine. These cells lost the fibrous and directional characteristics of the F-actin (Figure 3(a)). In addition, fluorescence intensity decrease comparing to the DMSO group gave a hint that brucine might downregulate the protein level of F-actin. Microtubule consists of tubulin proteins. We additionally investigated the effect of brucine on microtubule organization in MDA-MB-231 cells via immunofluorescence staining. It was illustrated that brucine caused significant disruption of microtubules (Figure 3(b)). Our results indicated that brucine caused the disruption of the cytoskeleton structures of MDA-MB-231 cells.

### 3.5. Brucine Inhibits the VM Formation

**Figure 4:**
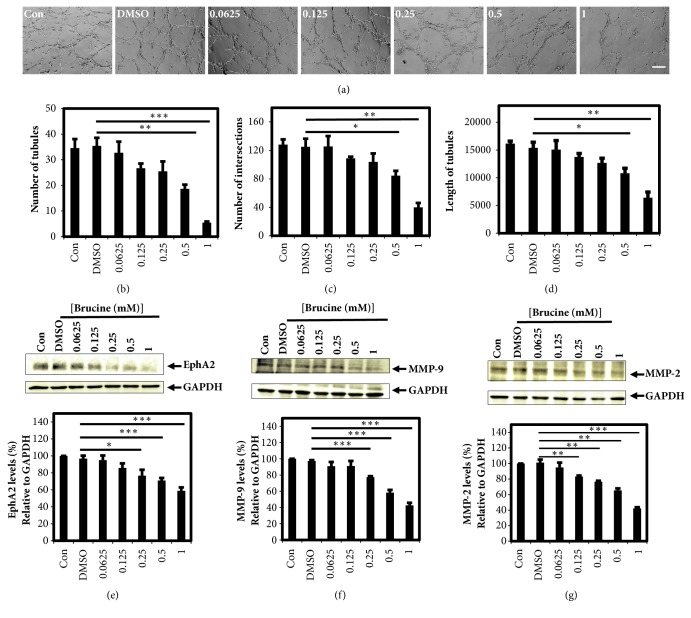
Brucine suppresses vasculogenic mimicry tube formation on matrigel with downregulation of VM-related protein expressions. The dose-dependent inhibitory effect of brucine on vasculogenic mimicry tube formation was observed under microscopy (a) and evaluated by the number of tubules (b) plus intersections (c) and the mean length of tubules (d) using ImageJ software. Western blot results and the corresponding densitometry analysis showed that brucine could reduce the protein expression levels of EphA2 (e), MMP-9 (f), and MMP-2 (g) with dose-dependent effect. The scale bar is of 200 *μ*m (mean ± s.e.m., n = 3, *∗*P < 0.05, *∗∗*P < 0.01, and *∗∗∗*P < 0.001.)

After MDA-MB-231 cells plated on matrigel in 24-well plates for 24 h, typical tubular networks emerged that represented VM (Figure S1). PAS staining showed VM formation in MDA-MB-231 cells (Figure S1). Brucine could significantly inhibit the VM formation with a dose-dependent effect in MDA-MB-231 cells (Figure 4(a)), and the inhibitory ability of VM formation induced by brucine was evaluated by the number of tubules (Figure 4(b)), number of intersections (Figure 4(c)), and mean length of tubules (Figure 4(d)).

### 3.6. Brucine Decreases the Protein Level of EphA2, MMP-9, and MMP-2 in MDA-MB-231 Cells

The protein levels of EphA2, MMP-9, and MMP-2 in the MDA-MB-231 cells were detected by western blot assays. GAPDH served as an internal reference. The results showed that brucine markedly reduced the levels of the EphA2 (Figure 4(e)), MMP-9 (Figure 4(f)), and MMP-2 (Figure 4(g)) proteins in a dose-dependent manner.

## 4. Discussion

In the present study, we provide the first evidence illustrating the role of brucine in the modulation of vasculogenic mimicry (VM) formation in the triple-negative breast cancer (TNBC) cell line MDA-MB-231. The inhibitory effect of VM is associated with the suppression of cell proliferation, migration and invasion, the aberration of cytoskeleton, and the downregulation of protein expressions of EphA2, MMP-9, and MMP-2.

As was shown in the previous reports, brucine could inhibit the tumor cell growth, migration, adhesion, invasion, angiogenesis, and/or metastasis and induce cell apoptosis and cell cycle arrest, holding a promise for tumor therapy [11, 12, 14–17]. The inhibitory effect of brucine on the proliferation of MDA-MB-231 cells was firstly illustrated here. We observed the decreased number of cells and the changes of cell morphology and shape (Figure 1(b)) after the treatment of different concentrations of brucine. The MTT assay results showed that brucine resulted in the dose-dependent inhibitory effect on MDA-MB-231 cell growth with the calculated IC50 1.172 mM. However, Annexin V/PI staining assay followed by FACS measurement indicated brucine only caused 4.27% apoptosis at the concentration of 1 mM (Figure 1(e)), which was consistent with the western blot assay results that increased cleaved caspase-3 only at the higher concentrations. In addition, cell invasion and migration analyses were also performed, the results of which showed that the invasive and migration abilities of MDA-MB-231 cells were negatively correlated with the increasing concentrations of brucine. Overall, it was inferred that brucine may have an important role in inhibiting MDA-MB-231 proliferation, thus providing a potential for VM suppression.

MDA-MB-231 cell line, one of triple-negative breast cancer cell (TNBC) lines, is aggressive and has a great tendency to metastasize to visceral organs [8]. Vasculogenic mimicry was detected in 35.8% of patients with TNBCs [8]. The highly invasive MDA-MB-231 cells could form VM, a channel structure lined by tumor cells rather than endothelial cells as a new pattern of tumor blood supply [7, 8, 10]. Here, we verified that MDA-MB-231 cells formed typical tubular structures with periodic acid Schiff (PAS) positive patterns of VM on matrigel, indicating that the aggressive MDA-MB-231 cells possessed VM formation capacity. In the following study, the effect of brucine on VM formation ability of MDA-MB-231 cells was observed under microscopy. And the inhibitory effect of brucine on VM formation was also evaluated by the number of tubules, number of intersections, and mean length of tubules. The results showed that brucine significantly inhibited the VM formation in a dose-dependent effect.

Vasculogenic mimicry was described as a process by Maniotis and colleagues for the first time, in which tumor cells developed highly patterned channel structure via rearranging of the F-actin cytoskeleton and matrix remodeling [3]. The previous studies also illustrated the relationship between vasculogenic mimicry and the change of cytoskeleton [3, 18–20]. Brucine's direct binding with CaM indicated that it might be novel leads for a CaM antagonist to potentially influence cell metabolism, cytoskeleton, and cell proliferation [21]. In this study, we provided the direct evidence demonstrating the disruption of the cytoskeleton induced by brucine. Herein, Alexa Fluor™ 488 Phalloidin with a specific affinity with F-actin was used to stain the F-actin cytoskeleton. Microtubule consists of tubulin proteins. Immunofluorescence assay using tubulin antibody was performed to indicate the microtubule structure. As was shown, the cells were treated with brucine lost the fibrous and directional characteristics of the F-actin (Figure 3(a)), in which the protein level of F-actin was also decreased. In addition, brucine caused significant disruption of microtubules (Figure 3(b)). In this study, it was demonstrated that the disruption of the cytoskeleton structures might contribute to the suppression effect of VM by brucine.

EPH receptor A2 (EphA2), MMP-9, and MMP-2 are responsible for the suppression effect on vasculogenic mimicry induced by brucine. The activated EphA2 relocalized to the cell membrane and phosphorylated drive the localization of FAK to new focal adhesion sites and subsequently activate PI3K, which thus triggers cell migration and VM channel formation [22]. In addition, the activated EphA2 proteins also directly activate PI3K, which then promotes the MMP precursor to form MMPs, ultimately promoting the cancer cell binding to form VM channels [22]. Among MMPs, MMP-2 and MMP-9 are two important enzymes, which are involved in the remodeling of extracellular matrix and are key mediators of invasion, metastasis, and tumor angiogenesis and facilitate VM formation [23, 24]. In the current study, it was found that the expression levels of EphA2, MMP-9, and MMP-2 were inhibited following brucine treatment, which was accompanied with a decrease in VM formation in MDA-MB-231 cells. These results indicated that the reduced expression levels of EphA2, MMP-9, and MMP-2 may be responsible for the inhibitory effect on vasculogenic mimicry induced by brucine in MDA-MB-231 cells. Future studies should confirm these observations in vivo.

## 5. Conclusions

Taken together, our results demonstrate that brucine inhibits the migration, invasion, and vasculogenic mimicry in human triple-negative breast cancer cell line MDA-MB-231, which might be through the disruption of cytoskeleton and downregulation of EphA2, MMP-9, and MMP-2. And these effects of brucine could potentially be exploited for new therapeutic strategies in tumor therapy.

## Data Availability

The data used to support the findings of this study are available from the corresponding author upon request.

## Supplementary Materials

Supplementary Materials Supplementary Figure S1: MDA-MB-231 cells formed typical tubular structures on matrigel. (a) 12 h after seeding MDA-MB-231 cells on matrigel, cell shape changes, including elongation and the formation of needle-like structures, were observed, and after 24 h typical tubular networks emerged. (b) The formed tubular structures could be stained by PAS. The scale bar is of 100 *μ*m.

## References

[1] El Hallani S., Boisselier B., Peglion F. (2010). A new alternative mechanism in glioblastoma vascularization: tubular vasculogenic mimicry. *Brain*.

[2] Folberg R., Hendrix M. J. C., Maniotis A. J. (2000). Vasculogenic mimicry and tumor angiogenesis. *The American Journal of Pathology*.

[3] Maniotis A. J., Folberg R., Hess A. (1999). Vascular channel formation by human melanoma cells in vivo and in vitro: vasculogenic mimicry. *The American Journal of Pathology*.

[4] Zhang S., Zhang D., Sun B. (2007). Vasculogenic mimicry: Current status and future prospects. *Cancer Letters*.

[5] Cascone T., Heymach J. V. (2012). Targeting the angiopoietin/Tie2 pathway: Cutting tumor vessels with a double-edged sword?. *Journal of Clinical Oncology*.

[6] Jain R. K., Duda D. G., Clark J. W., Loeffler J. S. (2006). Lessons from phase III clinical trials on anti-VEGF therapy for cancer. *Nature Clinical Practice Oncology*.

[7] Sun H., Zhang D., Yao Z. (2017). Anti-angiogenic treatment promotes triple-negative breast cancer invasion via vasculogenic mimicry. *Cancer Biology & Therapy*.

[8] Zhang D., Sun B., Zhao X. (2014). Twist1 expression induced by sunitinib accelerates tumor cell vasculogenic mimicry by increasing the population of CD133+ cells in triple-negative breast cancer. *Molecular Cancer*.

[9] Bergers G., Hanahan D. (2008). Modes of resistance to anti-angiogenic therapy. *Nature Reviews Cancer*.

[10] Jitariu A.-A., Cîmpean A. M., Ribatti D., Raica M. (2017). Triple negative breast cancer: The kiss of death. *Oncotarget *.

[11] Agrawal S. S., Saraswati S., Mathur R., Pandey M. (2011). Cytotoxic and antitumor effects of brucine on Ehrlich ascites tumor and human cancer cell line. *Life Sciences*.

[12] Saraswati S., Agrawal S. S. (2013). Brucine, an indole alkaloid from Strychnos nux-vomica attenuates VEGF-induced angiogenesis via inhibiting VEGFR2 signaling pathway in vitro and in vivo. *Cancer Letters*.

[13] Maes H., Van Eygen S., Krysko D. V. (2014). BNIP3 supports melanoma cell migration and vasculogenic mimicry by orchestrating the actin cytoskeleton. *Cell Death & Disease*.

[14] Hu K.-F., Kong X.-Y., Zhong M.-C., Wan H.-Y., Lin N., Pei X.-H. (2017). Brucine inhibits bone metastasis of breast cancer cells by suppressing Jagged1/Notch1 signaling pathways. *Chinese Journal of Integrative Medicine*.

[15] Li M., Li P., Zhang M., Ma F. (2018). Brucine suppresses breast cancer metastasis via inhibiting epithelial mesenchymal transition and matrix metalloproteinases expressions. *Chinese Journal of Integrative Medicine*.

[16] Li M., Li P., Zhang M., Ma F., Su L. (2014). Brucine inhibits the proliferation of human lung cancer cell line PC-9 via arresting cell cycle. *Zhongguo Fei Ai Za Zhi*.

[17] Li P., Zhang M., Ma W., Sun X., Jin F. (2012). Effects of brucine on vascular endothelial growth factor expression and microvessel density in a nude mouse model of bone metastasis due to breast cancer. *Chinese Journal of Integrative Medicine*.

[18] Fu D., He X., Yang S., Xu W., Lin T., Feng X. (2011). Zoledronic acid inhibits vasculogenic mimicry in murine osteosarcoma cell line in vitro. *BMC Musculoskeletal Disorders*.

[19] Xia Y., Cai X., Fan J. (2015). Rho kinase inhibitor fasudil suppresses vasculogenic mimicry of B16 mouse melanoma cells both in vitro and in vivo. *Molecular Cancer Therapeutics*.

[20] Zhou X., Gu R., Han X., Wu G., Liu J. (2017). Cyclin-dependent kinase 5 controls vasculogenic mimicry formation in non-small cell lung cancer via the FAK-AKT signaling pathway. *Biochemical and Biophysical Research Communications*.

[21] Ma L., Wang Z., Liu S., Song F., Liu Z., Liu S. (2013). Screening calmodulin-binding ligands using intensity-fading matrix-assisted laser desorption/ionization mass spectrometry. *Rapid Communications in Mass Spectrometry*.

[22] Zeng F., Ju R.-J., Liu L. (2015). Application of functional vincristine plus dasatinib liposomes to deletion of vasculogenic mimicry channels in triple-negative breast cancer. *Oncotarget *.

[23] Chang C., Werb Z. (2001). The many faces of metalloproteases: cell growth, invasion, angiogenesis and metastasis. *Trends in Cell Biology*.

[24] Chen L.-X., He Y.-J., Zhao S.-Z. (2011). Inhibition of tumor growth and vasculogenic mimicry by curcumin through down-regulation of the EphA2/PI3K/MMP pathway in a murine choroidal melanoma model. *Cancer Biology and Therapy*.

