# Supplementary material for: Brucine Suppresses Vasculogenic Mimicry in Human Triple-Negative Breast Cancer Cell Line MDA-MB-231
Source: Biomed Res Int. 2019 Jan 6;2019:6543230. doi: 10.1155/2019/6543230 (PMC6339755; doi:10.1155/2019/6543230)
Supplement: Supplementary Materials — Supplementary Figure S1: MDA-MB-231 cells formed typical tubular structures on matrigel. (a) 12 h after seeding MDA-MB-231 cells on matrigel, cell shape changes, including elongation and the formation of needle-like structures, were observed, and after 24 h typical tubular networks emerged. (b) The formed tubular structures could be stained by PAS. The scale bar is of 100 μm. [file 6543230.f1.doc]

Supplementary information


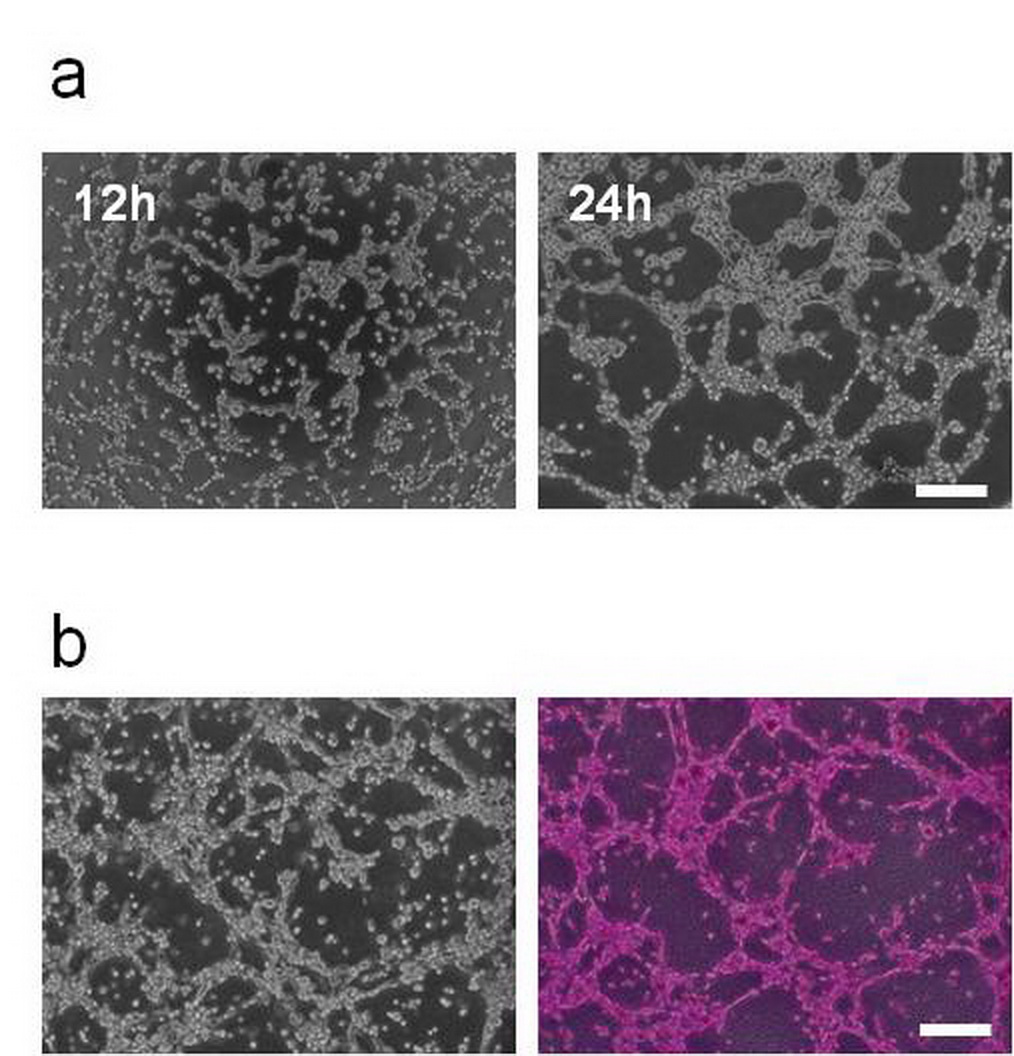


FIGURE S1：MDA-MB-231 cells formed typical tubular structures on Matrigel. (a) 12h After seeding MDA-MB-231 cells on Matrigel, cell shape changes, including elongation and the formation of needle-like structures were observed, and after 24 h typical tubular networks emerged. (b) the formed tubular structures could be stained by PAS. The scale bar is of 100 μm.
